# Retracted: A Rare Reason for Pelvic Pain in Pregnancy: Infectious Sacroiliitis

**DOI:** 10.1155/2020/2387532

**Published:** 2020-12-22

**Authors:** Case Reports in Medicine

*Case Reports in Medicine* has retracted the article titled “A Rare Reason for Pelvic Pain in Pregnancy: Infectious Sacroiliitis” [1] as it was found to contain text in the Introduction and Discussion sections that was taken from previously published articles, without citation [2, 3]. The sources are as follows:

- M. L. Moros, C. Rodrigo, A. Villacampa et al., “Septic shock in pregnancy due to pyogenic sacroiliitis: a case report,” *Journal of Medical Case Reports*, vol. 3, p. 6505, 2009, https://doi.org/10.1186/1752-1947-3-6505.
- D. Mahovic, N. Laktasic-Zerjavic, K. Tudor et al., “Pregnancy-related severe pelvic girdle pain caused by unilateral noninfectious sacroiliitis,” *Zeitschrift für Rheumatologie*, vol. 73, pp. 665–668, 2014, https://doi.org/10.1007/s00393-013-1323-6.

The authors did not approve the retraction, which was recommended by the editorial board.
